# Peripheral mechanisms of peripheral neuropathic pain

**DOI:** 10.3389/fnmol.2023.1252442

**Published:** 2023-09-14

**Authors:** Paola Pacifico, James S. Coy-Dibley, Richard J. Miller, Daniela M. Menichella

**Affiliations:** ^1^Department of Neurology, Feinberg School of Medicine, Northwestern University, Chicago, IL, United States; ^2^Department of Pharmacology, Feinberg School of Medicine, Northwestern University, Chicago, IL, United States

**Keywords:** neuropathic pain, peripheral neuropathic pain, single cell RNA seq, nociception, painful diabetic neuropathy

## Abstract

Peripheral neuropathic pain (PNP), neuropathic pain that arises from a damage or disease affecting the peripheral nervous system, is associated with an extremely large disease burden, and there is an increasing and urgent need for new therapies for treating this disorder. In this review we have highlighted therapeutic targets that may be translated into disease modifying therapies for PNP associated with peripheral neuropathy. We have also discussed how genetic studies and novel technologies, such as optogenetics, chemogenetics and single-cell RNA-sequencing, have been increasingly successful in revealing novel mechanisms underlying PNP. Additionally, consideration of the role of non-neuronal cells and communication between the skin and sensory afferents is presented to highlight the potential use of drug treatment that could be applied topically, bypassing drug side effects. We conclude by discussing the current difficulties to the development of effective new therapies and, most importantly, how we might improve the translation of targets for peripheral neuropathic pain identified from studies in animal models to the clinic.

## Introduction

Neuropathic pain is defined by the International Association for the Study of Pain (IASP) as “pain caused by a lesion or disease of the somatosensory nervous system” (Treede et al., 2008). A classification of syndromes associated with clinically relevant pain has been produced by a task force established by the IASP in collaboration with World Health Organization (WHO) representatives. This effort also resulted in the inclusion of chronic neuropathic pain (NeuP) diagnoses in the 11th revision of the WHO International Classification of Diseases and Related Health Problems (ICD) (Scholz et al., 2019; Treede et al., 2019). In addition, the IASP has updated the grading system for NeuP. Using this grading system, it is possible to define a diagnosis of possible, probable, or definite NeuP based on patient history, examination and confirmatory diagnostic tests (Finnerup et al., 2016; Colloca et al., 2017).

Neuropathic pain is broadly divided into central or peripheral neuropathic pain (PNP) (IASP, 1979; Bankar et al., 2018; Rumora et al., 2019). PNP arises from a damage or disease affecting the peripheral nervous system, including neuropathic pain associated with peripheral neuropathy (Loeser and Treede, 2008). PNP is associated with an extremely large disease burden and involves sensory dysfunctions, including spontaneous pain (shooting, burning or stabbing pain; “pins and needles” sensation) and increased evoked pain responses to mechanical and thermal stimuli. Despite the high prevalence of neuropathic pain (7%–8% of the general population) (van Hecke et al., 2014; Rice et al., 2016), current drugs available for treating PNP are only partially effective (Finnerup et al., 2015), and the long-term effects of these drugs, particularly opioids, are problematic (Finnerup et al., 2015; Vowles et al., 2015). The first-line recommended drugs (tricyclic anti-depressants, serotonin/noradrenaline reuptake inhibitors, and gabapentoids) produce limited relief in small subset of patients; indeed, one study reported that 4–8 patients must be treated for one patient to experience a 50% pain reduction compared with placebo (Finnerup et al., 2015). Opioids are among the less effective for treating neuropathic pain showing increased dilemmas linked to chronic use (Vowles et al., 2015; Jones et al., 2018). Considering the ongoing “opioid crisis” (Volkow and Blanco, 2021), it is crucial to understand neuropathic pain and develop novel, safer, non-addictive, and more valid treatments based on mechanistic targets specific to PNP.

One critical issue that prevents consistent, effective translation of preclinical studies to clinical efficacy is that there exists fundamental molecular and physiological differences in the biology of neuropathic pain between the rodent models currently used in preclinical studies and humans (Mogil, 2019). It is also not clear if these mechanisms depend on the characteristics underlying etiology; therefore, classifying neuropathic pain according to sensory phenotypes could provide more information about the pathophysiological mechanisms of these syndromes (Truini and Cruccu, 2016; Forstenpointner et al., 2018). Such mechanism-based approaches to the study of PNP might facilitate in adapting therapies for treating individual patients and could be helpful for drug development, allowing patients with selected phenotypes to power clinical trials (Truini and Cruccu, 2016; Vollert et al., 2017; Forstenpointner et al., 2021). Sensory phenotypes are defined using patient questionnaires for assessing pain quality (Bennett et al., 2007; Baron et al., 2012; Attal et al., 2018). In addition, a neurological examination can be used to evoke dynamic allodynia and diagnostic methodologies, such as quantitative sensory testing (QST); standard neurophysiological techniques, including electrophysiological studies, somatosensory and laser evoked potentials, microneurography and skin biopsy are used to determined sensory phenotypes (Truini and Cruccu, 2016; Colloca et al., 2017; Themistocleous et al., 2018). However, the stratification approach of QST has been questioned for (i) the inability to distinguish between painful and painless neuropathy, (ii) the discrepancy between experimentally evoked and spontaneous pain, and (iii) the potential discrepacy between the site of the sensory stimulation and the source of pain (Forstenpointner et al., 2021; Schmelz, 2021). Hence, although the strategy of differentiating patients according their sensory phenotypes is promising, stratification will most likely ultimately require multiple approaches, including not only QST, but also patient-reported questionnaires (Bouhassira et al., 2021), skin biopsy, and other more objective parameters or biomarkers, such as microneurography, evoked potentials and neuro-imaging combined with genetic screening (Calvo et al., 2019; Tracey et al., 2019; Egenolf et al., 2021).

While we are aware of the complex pathophysiological profile underlying PNP, ranging from primary terminal afferents in the skin (Feldman et al., 2017) to central areas in the spinal cord and brain that amplify and process nociceptive information (Colloca et al., 2017), in this review we will focus on peripheral mechanisms that trigger PNP, highlighting promising therapeutic targets that may be translated into disease modifying therapies for PNP.

### Peripheral neuropathic pain: developing novel pain treatments

The last 20 years have seen massive advances in our understanding of the neurobiology of pain and nociception, including the peripheral and central mechanisms underlying signal amplification and sensitization in nociceptive pathways accompanying pain-associated pathologies. Two approaches have been particularly effective in these efforts: the identification of genetic mutations that cause pain abnormalities in humans and the introduction of novel technologies applied to the study of painful neuropathy in animal models.

#### Genetic studies

Human genetic pain disorders have highlighted that the voltage-gated sodium channel α-subunits NaV_1.7_, NaV_1.8_ and NaV_1.9_ play a key role in the peripheral signaling of pain contributing to the generation of action potentials in sensory nociceptors (Akopian et al., 1996; Dib-Hajj et al., 1998; Bennett et al., 2019). Biallelic loss-of-function mutations in NaV_1.7_ have been identified in individuals insensitive or indifferent to a wide range of painful stimuli. On the other hand, NaV_1.7_ gain-of-function mutations are linked to debilitating chronic pain conditions, such as inherited erythromelalgia and paroxysmal extreme pain disorder (Dib-Hajj et al., 2008; Goodwin and McMahon, 2021). Moreover, pathogenic variants in NaV_1.7_, NaV_1.8_ and NaV_1.9_ have been found in up to 17% of patients with small fiber neuropathy (Faber et al., 2012a,b; Huang et al., 2014). Genetic studies can be instrumental in better understanding differing pain perceptions among patients with a similar neuropathy. For example, in the context of diabetic neuropathy, several different genetic approaches revealed rare NaV_1.7_ variants in 9% of patients affected by painful diabetic neuropathy (PDN); these patients reported more pain and higher sensitivity to mechanical stimuli on quantitative sensory testing compared to others in their cohort (Blesneac et al., 2018). Furthermore, in transgenic mouse models, the deletion of NaV_1.7_ in different neuronal subsets has demonstrated the critical contribution of this channel to different types of pain (Nassar et al., 2004). Similarly, the ablation of NaV_1.7_ in sensory and sympathetic neurons reduces mechanical hypersensitivity caused by spinal nerve transection, suggesting that a combined antagonism of NaV_1.7_ in both sensory and sympathetic neurons could be a potential successful strategy in treating pain states (Minett et al., 2012).

These observations further support the idea that NaV_1.7_ variants contribute to pain through their effects on the excitability of DRG neurons, where these channels play a key role in electrogenesis, and a great deal of effort has been invested in targeting NaV_1.7_ channel to develop analgesic processes (Dib-Hajj and Waxman, 2019). Drugs that reduce DRG neuronal hyperexcitability, such as sodium channel blockers (Eijkelkamp et al., 2012; Dib-Hajj et al., 2013), erase the excitability and increased [Ca^2+^]_i_, preventing not only neuropathic pain behavior but also the development of small-fiber degeneration, resulting in a potentially effective treatment for PDN. Generally, however, small-molecule inhibitors have failed to produce analgesic efficacy in phase II trials of PDN and post-herpetic neuralgia (Price et al., 2017; McDonnell et al., 2018). Despite these challenges, many companies are currently initiating discovery programs for the development of small molecule NaV_1.7_ inhibitors (Dib-Hajj and Waxman, 2019). For example, researchers at Genentech have used tarantula toxins to investigate the structural basis of NaV_1.7_ inhibition with the end goal of accelerating the development of next generation modulators (Xu et al., 2019). Some work proposes that NaV_1.7_ activity also modulates endogenous opioid peptide release, suggesting that the combined action of a NaV_1.7_ inhibitor and an opioid molecule may improve synergistic analgesia with fewer side effects (MacDonald et al., 2021). However, a publication has sought to disprove this mechanism suggesting that upregulation of opioid peptides play no role in the analgesic effects of NaV_1.7_ blockers (Bankar et al., 2018). Despite the promising genetic evidence, several selected NaV_1.7_ inhibitors have been discontinued prior or after phase II trials due to concerns about the lack of improvement of daily pain in patients with diabetic small fiber neuropathy. On the other hand, other molecules such as Vixotrigine (BIIB074), a voltage- and use-dependent Na^2+^ channel blocker, which was discontinued as a treatment for painful lumbosacral radiculopathy after phase II failure in 2018, is still in phase II trials for small fiber neuropathy (Kingwell, 2019; Faber et al., 2023). Recently, the selective NaV_1.8_ inhibitor VX-548, developed by Vertex Pharmaceuticals, began phase III clinical trials, including two randomized, double-blind, placebo-controlled studies to test the efficacy and safety of VX-548 for moderate to severe acute pain after surgeries for the removal of bunions (bunionectomy) and of excess abdominal fat (abdominoplasty) (Jones et al., 2023). The same molecule is also ongoing in phase II for treating PDN. Hence, specific Na^2+^ channel blockers are still of considerable interest for the development of novel analgesics.

Other potential routes to producing novel analgesics have also been suggested. It has been proposed that inhibition of the effects of the neurotrophin NGF may represent a way of treating pain in musculoskeletal conditions such as osteoarthritis (OA) (Wise et al., 2021). Activation of TrkA is known to produce rapid excitation of nociceptors through transactivation of TRPV1, and inhibition of the NGF-TrkA signaling pathway significantly reduces chronic pain caused by OA (Hefti et al., 2006; Lane et al., 2010). The involvement of TrkA signaling in pain has been suggested from the phenotype of patients affected by the rare genetic condition named Hereditary Sensory and Autonomic Neuropathy type IV (HSAN IV) (Indo et al., 1996). Loss of function mutations in TrkA have been found in HSAN IV affected individuals, leading to pain insensitivity, anhidrosis and intellectual disability (Indo, 2018). As observed in the humanized knock-in animal model of HSAN IV carrying the TrkA^R649W^ mutation, alterations in the phosphorylation state and membrane dynamics of TrkA result in a reduced pain sensation and compromised thermoregulation (Pacifico et al., 2023). These results suggest that the development of small molecules which reduce TrkA kinase activity could represent feasible analgesics candidates for pain treatment. Indeed, monoclonal antibodies against NGF have been shown to be effective in patients with OA associated pain (Lane et al., 2010). Unfortunately, this treatment also produced rapidly progressing OA in several previously unaffected joints underlying the complex role of TrkA signaling in the regulation of overall joint homeostasis (Hochberg et al., 2016; Katz, 2019).

Another genetic variant associated with neuropathic pain occurs in the gene encoding the transient receptor potential ankyrin 1 (TRPA1), a calcium-permeable non-selective cation channel, involved in peripheral neuropathic pain, such as diabetic neuropathy (Andersson et al., 2013; Hiyama et al., 2018). TRPA1 antagonism has been observed to decrease mechanical allodynia and hypersensitivity in a rodent model of diabetes induced by streptozotocin (Andersson et al., 2015). Moreover, TRPA1 can be activated by oxidative stress and glucose metabolism by-products, such as 4-hydroxy-2-nonenal (4-HNE) and methylglyoxal, which are elevated in diabetes, contributing to hyperalgesia (Trevisani et al., 2007). TRPA1 is also clearly involved in mechanical and cold allodynia evoked in mouse models of chemotherapeutic-induced peripheral neuropathy (CIPN), and thus also can represent a potential therapeutic candidate for CIPN. Indeed, anticancer drugs, such as oxaliplatin, paclitaxel, bortezomib, and the aromatase inhibitors exemestane, letrozole and anastrozole, can target TRPA1 eliciting hypersensitivity to mechanical and thermal stimuli (Gauchan et al., 2009; Trevisan et al., 2013).

In addition to genetic studies focusing on ion channels and transmembrane receptors, several genome-wide studies conducted in humans have been summarized in a systematic review and meta-analysis of genetic risk factors for neuropathic pain (Veluchamy et al., 2021). Further insights into the genetics of neuropathic pain have also been provided by the recently created Human Pain Genetics Database (HPGdb; https://humanpaingeneticsdb.ca/hpgdb/) (Meloto et al., 2018), a database including all genetic variants that are negatively or positively associated with neuropathic pain, which unsurprisingly include targets such as OPRM1 opioid receptors. Although this does not represent a novel target for pain, associated molecules such as G-protein receptor kinases (GRKs), which mediate the phosphorylation and desensitization of μ-opioid receptors (MOR), are considered another potential target for the treatment of peripheral neuropathies (Gamble et al., 2022).

Long non-coding RNAs (lncRNAs), have been recently reported as possible key modulators of gene expression networks, and have been proposed as a novel means of treating PNP conditions (Bali and Kuner, 2014; Wu et al., 2019). For example, the up-regulation of the lncRNA NONRATT021972 was reported to interact with two different ionotropic purinergic receptors, P2X3R in small-medium DRG neurons and P2X7R in satellite glia cells, both of which contribute to mechanical hypersensitivity (Liu et al., 2016; Peng et al., 2017). Indeed, P2X7R genetic variants linked to gain-of-function mutations have been found in individuals affected by PDN (Ursu et al., 2014). Additionally, lncRNAs and microRNAs (miRNA), which are found in several bodily fluids, have been reported to play a role in neuronal-immune communication, acting as long-distance messengers. By interfering with the physiological functions of miRNAs and lncRNAs, significant alterations have been observed in peripheral nerves and DRGs in diabetes, suggesting these molecules may play a key role in PNP (Wu et al., 2020).

Inflammatory processes have been increasingly suggested as being central players in the onset and maintenance of peripheral neuropathies. Patients with PDN have higher levels of pro-inflammatory mediator mRNAs and proteins in their blood, including inflammatory cytokines, suggesting a connection between inflammation and neuropathic pain (Uçeyler et al., 2007). For example, among hundreds of differentially expressed genes found in PDN patients, levels of the chemokine receptor CXCR4 are elevated (Hur et al., 2011). Similarly, the signaling cascade activated by the CXCR4 and its ligand, the chemokine CXCL12 (also known as stromal-derived factor 1), is fundamental in the generation of mechanical allodynia observed in the PDN mouse model (Jayaraj et al., 2018). The enhanced hyperexcitability triggered by CXCL12/CXCR4 signaling of DRGs of diabetic mice can be prevented by the selective chemogenetic inhibition of the NaV_1.8_-expressing DRG neurons, resulting in the rescue of mechanical allodynia and small-fiber degeneration (Jayaraj et al., 2018).

However, the big effort in translating genetic studies into effective therapies often finds some barriers. For example, (i) the reduced sample size due to the high cost of recruiting, phenotyping and genotyping the cohort, (ii) the heterogeneity of pain aetiology and phenotypic profiles of individuals within the cohort, (iii) the lack of common screening tools for neuropathic pain, can limit the advances toward developing effective pain treatments (Calvo et al., 2019). Despite the shortcomings of human genetic studies (Calvo et al., 2019), the identification of genetic risk factors for neuropathic pain continues to provide important clues as to the biological and physiological mechanisms involved in the onset and persistence of PNP. Furthermore, genetic studies have offered insights into the variability in pain perception in patients with similar neuropathies, for which the mutations discovered in NaV_1.7_ are important exemplars (Blesneac et al., 2018) in PDN patients, and in other chronic pain states. But human genetic studies alone are not sufficient. In recent years there has been a rapid development in technologies, such as optogenetics, chemogenetics and RNA-sequencing. In addition to the more traditional use of animal models of neuropathic pain and the studies on human genetic variations, such methods have allowed important progress in the understanding of mechanisms underlying neuropathic pain and the translation from animal data to humans.

Two powerful techniques have been recently employed for furthering our comprehension of nociceptors: optogenetics and chemogenetics. Using cell- or tissue- specific promoters, such as for example an Advillin-Cre driver mouse, or by adopting adeno-associated virus, optogenetic and chemogenetic techniques can target specific neuronal and non- neuronal cell types with limited off-target effects (Roth, 2016; Mickle and Gereau, 2018).

#### Optogenetic

Optogenetics can be used to modulate cellular activity by manipulating electrical currents generated by the activation of light-sensitive proteins called opsins (Boyden, 2015). Channelrhodopsin (ChR2), the most common “activating” opsin, originally sourced from algae, permeabalises neurons to cations when activated by blue light. In contrast, halorhodopsin allows anions to enter cells when exposed to yellow light producing neural inhibition. Due to cellular specificity and temporal precision, optogenetics has become a promising tool for the study of a wide-range of systems, including nociception and painful neuropathies in preclinical studies.

The conditional expression and activation of ChR2 using the viral vector AAV6 and the synapsin promoter in nociceptors expressing NaV_1.8_ or TRPV1 elicited nociceptive-like behaviors in rodents (Daou et al., 2013; Beaudry et al., 2017). In contrast, activation of the inhibitory proton pump archearhodopsin (Arch) in specific sensory neuron subpopulations, such as CGRPα and NaV_1.8_, attenuated pain responses in animal models of PNP or inflammatory pain (Daou et al., 2016; Cowie et al., 2018). Moreover, the selective activation of mechanoreceptors triggered by the blue light in animal models of neuropathic pain can also generate nocifensive responses (Dhandapani et al., 2018; Murthy et al., 2018). This indicates that selective expression of these channels in subtypes of DRG neurons could be instrumental in understanding the specific roles of these neurons in neuropathic pain.

Recently the application of optogenetic approaches revealed a novel role for non-neuronal cells in the skin in mediating PNP. Indeed, the direct optogenetic activation of keratinocytes can also generate nocifensive responses (Baumbauer et al., 2015; Moehring et al., 2018). However, despite the promising efficacy of optogenetics as a therapy for peripheral neuropathic pain conditions, the application of this technique to patients is just an initial step, point-out the necessity to optimize the opsin’s delivery, the safety of their expression and the development of handy light-generating tools (Mickle and Gereau, 2018; Murthy et al., 2018).

#### Chemogenetics

Among several classes of chemogenetically engineered proteins, Designer Receptors Exclusively Activated by Designer Drugs (DREADDs) are the most widely used in neuroscience studies (Armbruster et al., 2007; Roth, 2016). DREADD receptors are engineered via the molecular evolution of muscarinic receptors resulting in their high-affinity, selective binding, and activation by the synthetic ligand clozapine-N-oxide (CNO). DREADDs are G protein coupled receptors (GPCRs), and CNO binding to DREADDs activates the classic GPCR signaling pathways Gq, Gi and Gs in different cell types. By administering CNO, either locally or systemically, DREADDs expressed under the control of cell-type specific promotors can be selectively activated in the desired cell type with minimal off-target impact. Similarly to optogenetics, engineered DREADDs can have activating or inhibiting properties based on coupling to Gq- or Gi-type G proteins. hM3Dq, one of the first GPCR-based DREADDs, has been extensively used to define sensory neuron features. The selective expression of hM3Dq DREADD in Mrgprd+ sensory neurons elicited itch behavior in rodents (Liu et al., 2012; Hill et al., 2022). In contrast, chemogenetic inhibition of NaV_1.8_-neurons, mediated by the expression of hM4Di, reverses the mechanical allodynia induced by the surgical destabilization of the medial meniscus, a model of progressive osteoarthritis (Miller et al., 2017). In a mouse model of PDN, it has been shown that hM4Di expressed in NaV_1.8_-positive neurons was able to modulate their neuronal excitability, reversing mechanical allodynia and small fiber degeneration (Jayaraj et al., 2018). Discovering naturally occurring GPCRs expressed in the same subsets of neurons that recapitulate these effects may therefore provide novel targets for inhibiting nociceptor excitability. Hence, both optogenetics and chemogenetics represent promising tools for identifying novel neuronal targets for treating neuropathic pain (Bennett et al., 2007).

The relatively high success rates of trials for GPCR ligands has led to the approval by the FDA of 481 GPCR-targeting drugs, and many GPCR-targeted molecules are being currently assessed in clinical trials for diabetes and obesity (Hauser et al., 2017). Neuronal functions can be modulated by GPCRs both directly, through the modulation of ion channels, and indirectly, by phosphorylation induced by secondary messengers, thereby controlling the selective flux of ions across the neuronal cell membrane. For example, in the spinal cord, the blocking of the N-type Voltage-gated Ca^2+^ channels (VGCCs) by activating GPCRs blocks nociceptive transmission, leading to a profound analgesia in animals and humans (Wang et al., 2000).

Despite the great potentiality of DREADDs, scientists have to always consider the limitations linked to the use of chemogenetic approaches. Indeed, clozapine, a product of CNO metabolism, is an antipsychotic drug that can act on other endogenous receptors causing undesirable side effects (Gomez et al., 2017). This main limitation might be constrained by administering CNO to control samples non-DREADD-expressing or employing other selective DREADD-agonist compounds, such as perlapine (Mahler and Aston-Jones, 2018).

## Potential therapeutic interventions to treat neuropathic pain

Genetic, *in vivo* Ca^2+^ imaging, chemogenetic and optogenetic studies applied to preclinical models of neuropathic pain have demonstrated the crucial importance of DRG neuron hyperexcitability and associated increased intracellular Ca^2+^ concentrations in inducing the axonal degeneration underlying neuropathic pain associated with painful neuropathies. The complex nature of PNP has encouraged investigation of the diverse mechanisms that may underly this condition. Here, we discuss three different novel approaches:

### Targeting DRG neuron excitability

Neuropathic pain is linked to hyperexcitability of neurons in pain pathways in the absence of proper stimuli; DRG nociceptors are the main mediators of this phenomenon (Zhang et al., 1999; Latremoliere and Woolf, 2009). Patients present with abnormal spontaneous action potentials in the nerve terminals of C-fiber nociceptors that can be recorded with microneurography, a technique particularly suited for recording from C-fibers (Orstavik and Jørum, 2010; Serra et al., 2015). Diabetic patients (Ørstavik et al., 2006) and animal models of PDN (Bierhaus et al., 2012; Andersson et al., 2013) show hyperexcitability of sensory neurons, including spontaneous activity of DRG nociceptor axons (Bierhaus et al., 2012; Serra et al., 2015). In line with these observations, decreasing the hyperexcitability of DRG nociceptors, identified by the Na^2+^ channel Na_v_1.8, which is expressed by 90% of nociceptors (Shields et al., 2012), not only reversed mechanical allodynia in the well-established high-fat diet (HFD) mouse model of PDN (Obrosova et al., 2007), but also reversed small-fiber degeneration (Jayaraj et al., 2018).

Increased membrane excitability is associated with increased calcium influx into nociceptors, leading to a higher concentration of intracellular calcium. Indeed, hyperexcitability of Na_v_1.8 DRG neurons was followed by an increase of intracellular calcium level ([Ca^2+^]_i_) in the HFD mouse model of PDN (Jayaraj et al., 2018). Sustained increase [Ca^2+^]_i_ is a crucial factor in the signaling pathways leading to axonal degeneration (Wang et al., 2012) in both the central (Coleman and Hugh Perry, 2002) and peripheral nervous systems (Lehning et al., 1996; Vargas et al., 2015). Higher [Ca^2+^]_i_ can lead to DRG neurite degeneration in a genetic mouse model of small-fiber neuropathy (Estacion et al., 2015). Increased [Ca^2+^]_i_ might be involved in axonal degeneration by altering mitochondrial function (Bernardi and Rasola, 2007), including changes of mitochondrial calcium homeostasis (Rasola and Bernardi, 2007). Morphology and dynamics of mitochondria can be also affected by calcium levels through the regulation of dynamin-related protein 1 (Drp1) phosphorylation (Cribbs and Strack, 2007; Han et al., 2008). Mitochondria are involved in the axonal pathology observed in PDN (Vincent et al., 2010, 2011). Down-regulation of mitochondrial respiratory chain complex proteins (Chowdhury et al., 2012) and reduced respiratory chain activity (Chowdhury et al., 2010) have been observed in DRG neurons from mouse models of type II diabetes. Moreover, the morphology and localization of mitochondria are changed in animal models of PDN and in PDN patients (Lauria et al., 2003; Edwards et al., 2010; Vincent et al., 2010, 2011). The genetic model of type-2 diabetes, the db/db mice, reported elevated calcium levels in DRG neurons (Huang et al., 2002) and altered mitochondrial calcium homeostasis (Fernyhough and Calcutt, 2010), as well as changes in the morphology and trafficking of mitochondria (Edwards et al., 2010; Rumora et al., 2019) and increased fission (Edwards et al., 2010). All these mechanisms are indeed fundamental for neuronal function (Court and Coleman, 2012; Chan, 2020). Ongoing modifications in inter-mitochondrial networks, as well as shape, size, connectivity, trafficking, and activity of mitochondria belong to dynamic mechanisms of mitochondria (Chan, 2020). Mitochondrial morphology is defined by the balance between the opposing forces of fusion and fission (Sabouny and Shutt, 2020). Intramitochondrial [Ca^2+^]_i_ might play a key function in the processes regulating dynamics and morphology (Cribbs and Strack, 2007; Han et al., 2008). Mitochondrial calcium influx is allowed by the mitochondrial calcium uniporter (MCU), a selective calcium channel that promotes transport of calcium across the inner membrane when intracellular calcium concentration [Ca^2+^]_i_ reaches the particular cellular “set point” (Baughman et al., 2011; Stefani et al., 2011). In myocardial ischemia/reperfusion (I/R) injury (Zhao et al., 2015) has been observed the upregulation of MCU expression and the increase of mitochondrial fission (Zhao et al., 2015; Guan et al., 2019). In I/R injury, the pharmacological block of MCU reduces myocardial infarction by affecting mitochondria fission (Zhao et al., 2015). Similar mechanisms appear to be involved in axonal degeneration and mechanical allodynia in the well-established HFD mouse model of PDN (Jayaraj et al., 2018). Indeed, the selective deletion of MCU from nociceptors, by preventing calcium entry into the mitochondria, recovered mitochondrial morphology and dynamics, inhibited axonal degeneration, and rescued mechanical allodynia in the HFD mouse model of PDN (George et al., 2022). Hence, we suggest that targeting the increased calcium influx into nociceptor mitochondria mediated by MCU may be a promising strategy to disease-modifying therapies for patients suffering from PNP such as PDN.

#### Targeting axonal degeneration

The axon degeneration that occurs in many peripheral neuropathies is poorly understood. Thus, defining these underlying mechanisms could present novel therapeutic targets for axon preservation and neuropathic pain prevention (Simon and Watkins, 2018). Recent genetic and biochemical studies have identified several molecular mechanisms driving axonal degeneration. One study highlighted the critical role for the Sterile Alpha and TIR Motif containing 1 (SARM1) gene as the first compelling axonal-specific target for therapeutic intervention (DiAntonio, 2019; Krauss et al., 2020). Nicotinamide mononucleotide adenylyl transferase (NMNAT2) is a fundamental protein that supports axonal integrity, and damages that typically alter the axon’s cytoskeleton reducing the axonal transport can lead to axonal degeneration. Decreased levels of NMNAT2 causes the accumulation of the nicotinamide mononucleotide (NMN) and the activation of SARM1 (Sasaki et al., 2016; Walker et al., 2017). SARM1 activation leads to a rapid and almost total depletion of axonal NAD^+^ followed by the decrease of ATP, leading to a bioenergetic crisis within axons and a conseguent axonal collapse (Gerdts et al., 2015; Essuman et al., 2017), that release neurofilament light chain protein (Vial, 1958), which can be detected in serum (Disanto et al., 2017). This suggests that SARM1 may be a potential target for axonal preservation and that neurofilament light chain may be a translatable biomarker allowing the development of a new group of therapeutical molecules for the treatment of axonopathies.

While the link between SARM1 and axon degeneration was initially observed in response to axotomy (Gerdts et al., 2013, 2015; Tian et al., 2020), recent studies have shown that SARM1 can mediate axonal loss in animal models of chemotherapy-induced peripheral neuropathy (Osterloh et al., 2012; Geisler et al., 2019; Cetinkaya-Fisgin et al., 2020), in models of diabetic neuropathy (Cheng et al., 2019), and neuropathy associated with metabolic syndrome (Turkiew et al., 2017; Cheng et al., 2019). Furthermore, SARM1 is reported to play a critical in axonal degeneration caused in human-derived sensory neurons (Chen et al., 2021). Recently, novel upstream molecular mechanisms that trigger SARM1 activation have been proposed (Rice et al., 2016). On the other hand, small molecules inhibiting SARM1 promote the rescue of damaged axons that would otherwise degenerate (Hughes et al., 2021), and inhibiting SARM1 pharmacologically might protect axon organization and function in paclitaxel-induced peripheral neuropathy (Bosanac et al., 2021).

Microtubules (MTs), a prominent component of axonal cytoskeletons, play a key role in several cellular functions, including cell division, structure, and intracellular transport. MTs undergo polymerization and depolymerization of α/β tubulin heterodimers, resulting in the “dynamic instability” process that involves the binding, hydrolysis, and the exchange of GTP molecules on the β-tubulin monomers (Mitchison and Kirschner, 1984). Mutations of β-tubulin can affect vesicular axonal transport. The expression of β-tubulin class III (TUBB3) is maintained at elevated levels in the adult peripheral nervous system, suggesting a fundamental role for these proteins for the maintenance of peripheral motor and sensory neurons. Indeed, individuals with missense mutations in TUBB3 exhibit a progressive loss of peripheral axons that degenerate in sensory-motor neuropathy (Tischfield et al., 2010). The axonal degeneration of sensory neurons observed in patients can be explained by the impaired transport of vesicles and mitochondria in DRG expressing E410K or D417H mutations in TUBB3 (Niwa et al., 2013). In addition to β-tubulin, KIF1A, the member of the kinesin-3 family involved in the transport of synaptic vesicles, has been reported to play an essential role in axonal neuropathy and several neurological symptoms observed in patients. In DRGs, KIF1A mediates the transport of vesicles containing TrkA in DRG neurons from the cell body to the axon tip, and also facilitates the membrane expression of capsaicin receptor TRPV1 (Tanaka et al., 2016). Furthermore, mice lacking KIF1A (*Kif1a*^+/−^) die prematurely, and exhibit a strong reduction of synaptic vesicle density at nerve endings and an accumulation in the DRG soma (Morikawa et al., 2022), features associated with the progressive sensory impairment observed in mice (Tanaka et al., 2016). The link between KIF-family members and axonal degeneration is also observed in Hereditary Sensory and Autonomic Neuropathy Type II (HSAN2), causing progressively reduced sensation of pain, temperature, and touch, and in the Hereditary Spastic Paraplegia or typical Charcot–Marie–Tooth disease type 2 (CMT2) (Nair et al., 2023). Mutations in KIF1A or KIF5A are found in individuals affected by HSAN2 and CMT2, respectively, highlighting the unique role of these molecules in anterograde transport of mitochondria in axons (Morikawa et al., 2022). KIF26A, another member of the kinesin superfamily of proteins, is expressed in peripheral nociceptive neurons and involved in the mediation of sensory stimuli. The complete loss of KIF26A in *Kif26a*^−/−^ mice results in prolonged responses to painful stimuli caused by sustained calcium transients and neuronal excitation in primary DRG *Kif26a*^−/−^ cultures and in the overdevelopment of DRG axons. The inhibition of SFK-FAK signaling transduction, by known KIF26A interactors, can reverse the critical phenotype found in *Kif26a*^−/−^ mice (Wang et al., 2018).

#### Targeting cutaneous nociceptors

A hallmark of painful peripheral neuropathy is the degeneration of small fibers, a “dying back” axonopathy that interests the smallest axons of the peripheral nervous system, typically the DRG nociceptor afferents (Lauria et al., 2012) that extend to innervate the skin. The skin is densely innervated with a complicated network of molecularly distinct cutaneous nerve subtypes (Usoskin et al., 2015). In the last decade, the full cellular diversity and functional heterogeneity of cutaneous sensory afferents has begun to be determined (Chiu et al., 2014; Li et al., 2016). These discoveries have advanced our understanding of the pathogenic mechanisms underlying axonal degeneration in painful peripheral neuropathy and could lead to promising disease modifying therapeutics for PNP.

The classification of early sensory nerve was originally based on factors like size, speed of impulse conduction, and function. Among these nerves, there are thinly myelinated Aδ-fibers responsible for carrying thermal, mechanoreceptive (pressure), and acute nociceptive (pain) signals. C fibers (~70%), which are small and unmyelinated, are involved in the transmission of painful inputs, temperature, and itch, propagating impulses at a slower rate and in a more sustained manner compared to Aδ fibers (Willis, 2004; Fang et al., 2005; Ruscheweyh et al., 2007). C fibers have been traditionally classified into two subsets, peptidergic (PEP) and non-peptidergic (NP). Peptidergic C fibers can release neuropeptides, such as substance P (SP) and calcitonin gene-related peptide (CGRP), and express TrkA, the main receptor for nerve growth factor (NGF, see above). In contrast, non-peptidergic C-fibers bind to isolectin B4 (IB4) and express the ATP-binding purinergic receptor P2X3 in rodents (Basbaum et al., 2009). In the last decade, several studies have led to an increased understanding of the full cellular diversity and functional heterogeneity of cutaneous sensory afferents (Chiu et al., 2014; Li et al., 2016). For example, unmyelinated non-peptidergic C fibers innervating the skin and expressing Mrgprd are involved in the response to mechanical noxious stimuli in both rodents (Rau et al., 2009) and humans (Dong et al., 2001), also through a potential communication with non-neuronal epidermal cells (Zylka et al., 2005), suggesting that Mrgprd downregulation can reduce the mechanical hypersensitivity in inflammatory pain (Cavanaugh et al., 2009) and in PDN. On the other hand, also targeting A-fibers, usually expressing neurofilament heavy chain (Nefh) (Usoskin et al., 2015), might be a successful strategy to treat mechanical pain in anticancer-induced peripheral neuropathy (Xu et al., 2015).

**Figure 1:**
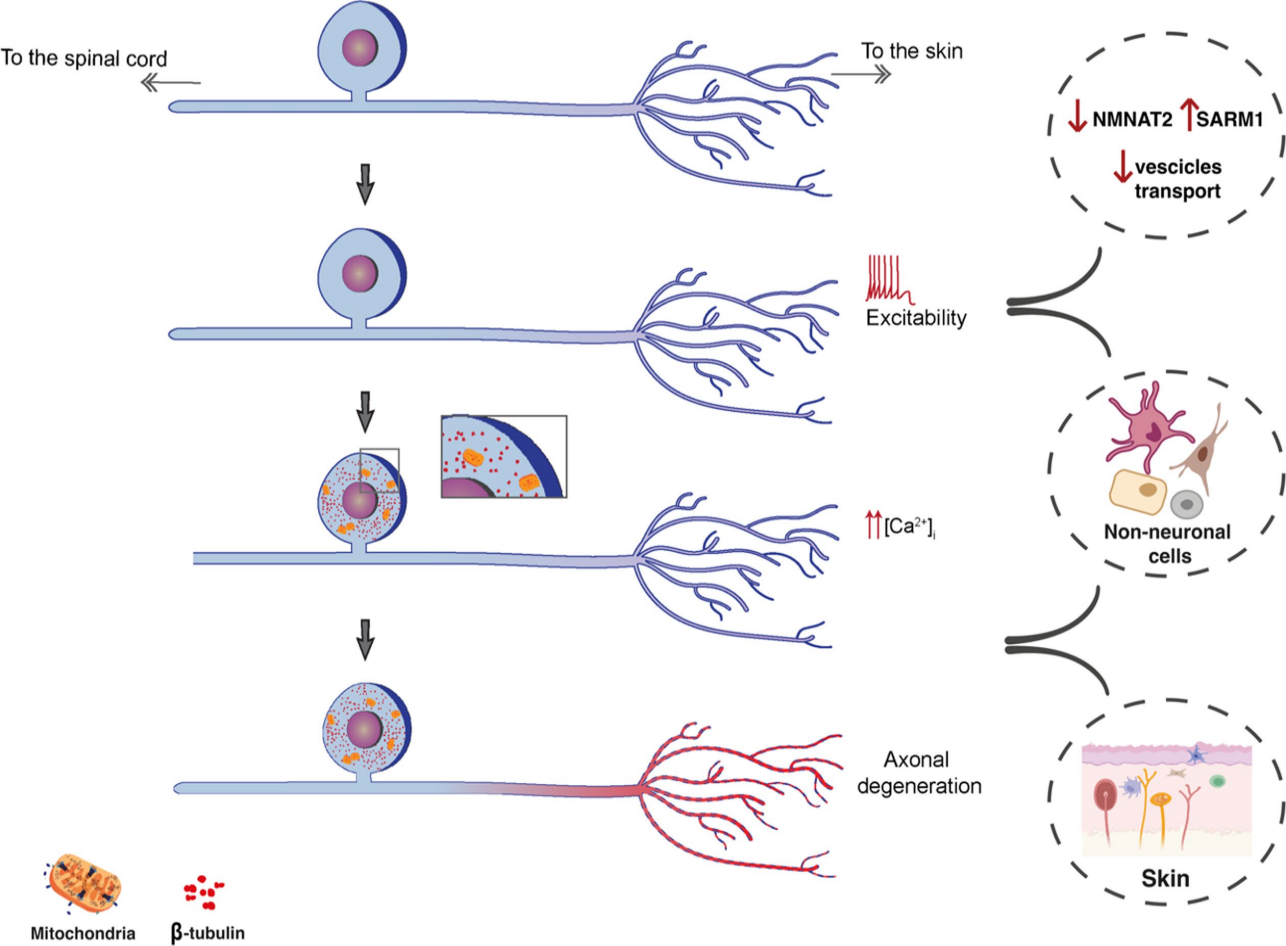
Schematic representation showing the possible workflow of peripheral mechanisms of NeuP. The increased excitability, leading to increased calcium influx culminates with axonal degeneration. Among potential players, alterations in axonal protein transports (SARM1 and KIFs) and the involvement of non-neuronal cells both in DRGs and the skin can contribute to the degeneration of nociceptive fibers and neuropathic pain.

Recent findings on human DRGs have highlighted many differences between rodents and humans, showing, for example, that P2X3 mostly colocalises with CGRP in human nociceptors (Shiers et al., 2020; Middleton et al., 2021). By applying single cell transcriptomics, it has become evident that DRG neurons show a remarkable level of heterogeneity, suggesting that their functionalities may be finely tuned in accordance with their phenotype (Figure 1).

## Cutaneous interactions in peripheral neuropathic pain

**Figure 2:**
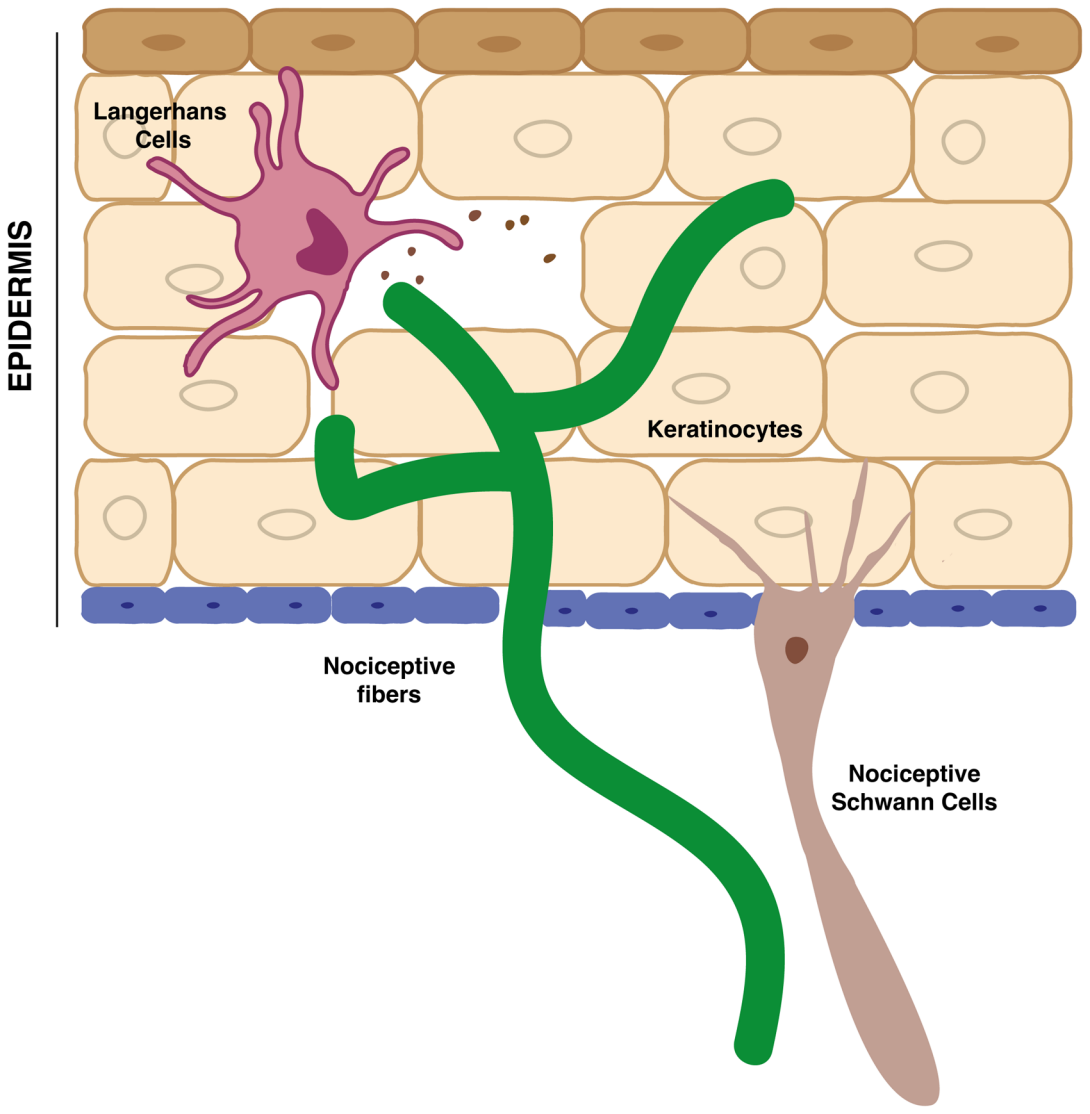
Interaction between terminal nociceptive fibers in the epidermis and non-neuronal cells, such as keratinocytes, Langerhans cells and nociceptive-Schwann cells.

Being our largest sensory organ, the skin is a remarkable mosaic of defined sensory areas in which terminal epidermal nerve afferents from DRG neurons communicate with a diverse array of distinct cell types (Figure 2).

Keratinocytes (KCs), the most abundant cell type population in the epidermis, differentiate from a single lineage to become the building blocks of the epidermis. The regenerative capacity and constant turnover of the epidermis results from the ability of KCs to proliferate, migrate and differentiate across the epidermis, releasing lipids, keratin and maintaining skin homeostasis. However, the importance of KCs is not just limited to providing the structural backbone of the skin; they also participate in a wide range of cellular processes in health and pathological conditions, including nociception, itch, inflammation, and metabolic conditions. KC distribution in different epidermal layers suggests that these cells can play distinct roles in the communication with nerve afferents. Indeed, a variety of neuronal subpopulations terminate in the epidermis (Colloca et al., 2017; Handler and Ginty, 2021), providing an opportunity for both gap junctions and synapse-like contacts (Churko and Laird, 2013; Woo et al., 2015), as well as indirect crosstalk between keratinocytes and nerve afferents (Finnerup et al., 2016). However, the complex heterogeneity of KCs further complicates our understanding of the extent of KC-nerve communication, and the specific role of KCs in nerve degeneration under pathological conditions has not been widely investigated. KCs are involved in the detection of touch stimuli in the skin, participating in the transmission of mechanical information related to pressure and brushing (Löken et al., 2009; Moehring et al., 2018; Mikesell et al., 2022). Indeed, under physiological conditions, the optogenetic inhibition of KCs, based on the expression of Archaerhodopsin-3 (Arch) in K14-expressing epidermal cells, which represent the basal-layer progenitor keratinocytes, reduces the response to mechanical non-noxious stimuli, suggesting their functional role in touch (Moehring et al., 2018). The crosstalk between KCs and nerve terminals can be promoted by the activation of the mechano-transducer PIEZO1 and/or by the release of ATP molecules via ATP-P2X4 signaling pathway (Moehring et al., 2018; Mikesell et al., 2022). Contrary to PIEZO1 knock-out mice that are unresponsive, intraplantar injection of Yoda1, a specific PIEZO1 agonist, elicits pain behavior in rodents and causes C-fiber hypersensitivity, suggesting that keratinocyte PIEZO1-activation mediates touch sensation (Mikesell et al., 2022).

Recent single-cell transcriptomics on human skin cells (Cheng et al., 2018; He et al., 2020; Wang et al., 2020) have highlighted potentially novel pharmacological targets (Theocharidis et al., 2022). For example, using single cell-RNA sequencing of the human neonatal epidermis, the group of Dr. Scott Atwood has investigated the dynamic nature of KCs and their hierarchical distribution in the epidermis (Wang et al., 2020). In addition to KCs, fibroblast heterogeneity has also been explored in human skin. Indeed, Tabib and colleagues have found different fibroblast populations in healthy human skin, characterizing two major groups, defined by SFRP2 and FMO1 genes and involved in regulating matrix deposition and inflammation (Tabib et al., 2018). Human epidermal cells isolated from pathological skin, including patients with psoriasis and eczema, have shown that keratinocytes mediate inflammatory responses in skin disorders by the activation of NF-kB pathway that can be blocked by the expression of ABIN1 and A20, both inhibitors of NF-kB (Harirchian et al., 2019). In addition to higher levels of keratins (Cheng et al., 2018), psoriatic human skin also shows an enrichment in dendritic cells (Cheng et al., 2018) that could be linked to itch sensation carried by C-fibers (Komiya et al., 2020). However, a comprehensive understanding of the heterogeneity of different cell populations in the skin and their role in pathological conditions remains unclear.

Langerhans cells (LCs), discovered by the German physician Pal Langerhans in 1868, are a small population of epidermis-resident cells. LCs were initially, albeit erroneously, classified as nerve cells in the epidermis, but their complete characterization has evolved over time. Classically, LCs have been viewed as being exclusively involved in inflammatory responses, acting as specialized antigen-presenting cells capable of a low rate of local, self-renewal capacity and migration to lymph nodes upon activation. However, their morphological and functional features indicate that LCs have a macrophage-related lineage rather than being a subset of dendritic cells (Satpathy et al., 2012; Kaplan, 2017). Interestingly, murine LC expression has been observed in many neuropathic pain conditions, such as after sciatic nerve transection or in chronic constriction of the sciatic nerve (Hsieh et al., 1996; Lindenlaub and Sommer, 2002). Both studies report that the denervation caused by sciatic nerve transection or chronic constriction injury, respectively, led to an increased number of LCs in the epidermis, correlating with a higher thermal and mechanical pain sensitivity in injured rodents. Interestingly, quantification using Langerin/CD207^+^ cells, an established marker used to label LCs in the epidermis, using skin biopsy samples of patients with diabetic small fiber neuropathy, revealed higher counts of LCs, also indicating a negative correlation between the number of LCs and epidermal innervation (Casanova-Molla et al., 2012). Comparable results have also been observed in a mouse model of PDN, the db/db knock-out mice, suggesting a potential role of these cells in the maintenance of mechanical allodynia (Dauch et al., 2013). Though LCs are involved in the neuroimmune response of the epidermis, their potential interaction with sensory fibers remains uninvestigated.

The communication between nerve terminals and non-neuronal cells in the skin has also been explored by Rinwa et al. (2021) who showed that a specialized cutaneous glial cell type conveys noxious thermal and mechanical sensitivity via a mesh-like network of processes at the subepidermal border of the skin. These “nociceptive Schwann cells” (NSCs) are found in both human and mouse skin and are located at the dermal-epidermal junction. Since their projections form contacts with terminal afferents of nociceptors in the epidermis, the authors discovered that the ablation of NSCs resulted in the retraction of nociceptive fibers and, consequently, mechanical, cold, and heat hyperalgesia. The ablation of peptidergic TRPV1^+^ fibers led to a similar phenotype in rodents to those observed in NSC ablated mice, suggesting that the mutual dependence of the epidermal nerve and the Schwann cell processes is critical for epidermal nerve innervation and transmission of nociceptive stimuli (Rinwa et al., 2021). Exploring the communication between non-neuronal cells and nerve terminals in the skin is a crucial step for developing novel therapeutic strategies to treat pain conditions (Lowy et al., 2021).

Overall, our improved understanding of the details of communication between nerve terminals and non-neuronal cells in the skin could well be the basis for new therapeutic approaches that could be applied topically bypassing drug side effects associated with systemic interventions.

## Limitations of preclinical research in rodent models: lost in translation

Despite the substantial knowledge gained from animal models over the last 70 years on the neurological systems subserving pain, there has been an absence of therapeutic progress for human patients. Many factors limit the translation of pain candidates previously identified from studies in animal models to humans, notably the significant behavioral and transcriptomic differences between the two species. Here, we will discuss both.

### Behavioural readouts

The evaluation of PNP in preclinical models requires indirect behavioral readouts as a surrogate of the pain experience. This can substantially limit the capacity of animal models to be predictive clinical efficacy (Patel et al., 2017; Sexton et al., 2017; Yekkirala et al., 2017; Mogil, 2019) due to significant differences in pain behaviors between humans and mice. In animal models, one of the most common behavioral outcome is measured as reflex withdrawal threshold evoked by thermal or mechanical stimuli aimed to test their hypersensitivity (Mogil, 2009; Deuis et al., 2017). On the other hand, pain evaluation in patients relies on questionnaires based on conscious pain experience, usually associated to a continuous pain sensation known as “spontaneous pain” and mediated by pathophysiological mechanisms different from those associated to “evoked pain.” Developing novel techniques and approaches aimed at evaluating spontaneous pain in rodents requires a significant but necessary effort, and medications intended to relieve ongoing pain in humans should be tested in appropriate preclinical assays for ongoing or spontaneous pain rather than relying solely on evoked pain.

To address these obstacles, experimental endpoints in our pre-clinical and early-stage clinical investigations need to align. In preclinical studies in mice, alongside rigorous randomization, blinding, experimental design reproducibility, and a consideration of biological variability and sex differences (Mogil, 2012; Mifflin and Kerr, 2014; Sorge et al., 2015; Sorge and Totsch, 2017), it would be helpful to use behavioral assays to monitor naturalistic behaviors, such as wheel running, gait change, home cage activities, conditioned place preference or aversion tests, where one can measure whether a drug reduces aversion or induces preference in the context of pain (He et al., 2012). An additional shortcoming that could affect the success of pre-clinical studies is represented by the exclusive use of male animals in the majority of experiments. Indeed, if the exclusion of female subjects due to the large fluctuations in hormones (Becker et al., 2005) might reduce the variability within the group, on the other side *“basic scientists are shirking their responsibilities to half of the human population”* (Mogil and Chanda, 2005). We can now consider the integration of female subjects in experiments and utilize novel technologies to assess spontaneous pain in mice, such as capturing paw kinematics during pain behavior with high-speed videography and automated paw tracking using machine learning optimization (Langford et al., 2010; Abdus-Saboor et al., 2019; Jones et al., 2020). Another endpoint could be the use of ultrasonic vocalization, inaudible sounds used by rodents to communicate and share emotional states (Premoli et al., 2021). Despite the value of these vocalizations, especially for neurodevelopmental studies, it is still unclear if these can be used as viable parameters for evaluating the response to noxious stimuli (Wallace et al., 2005). Overall, careful consideration of behavioral endpoints in preclinical studies and alignment of these with early human studies may help to better predict translational potential of a new target.

**Figure 3:**
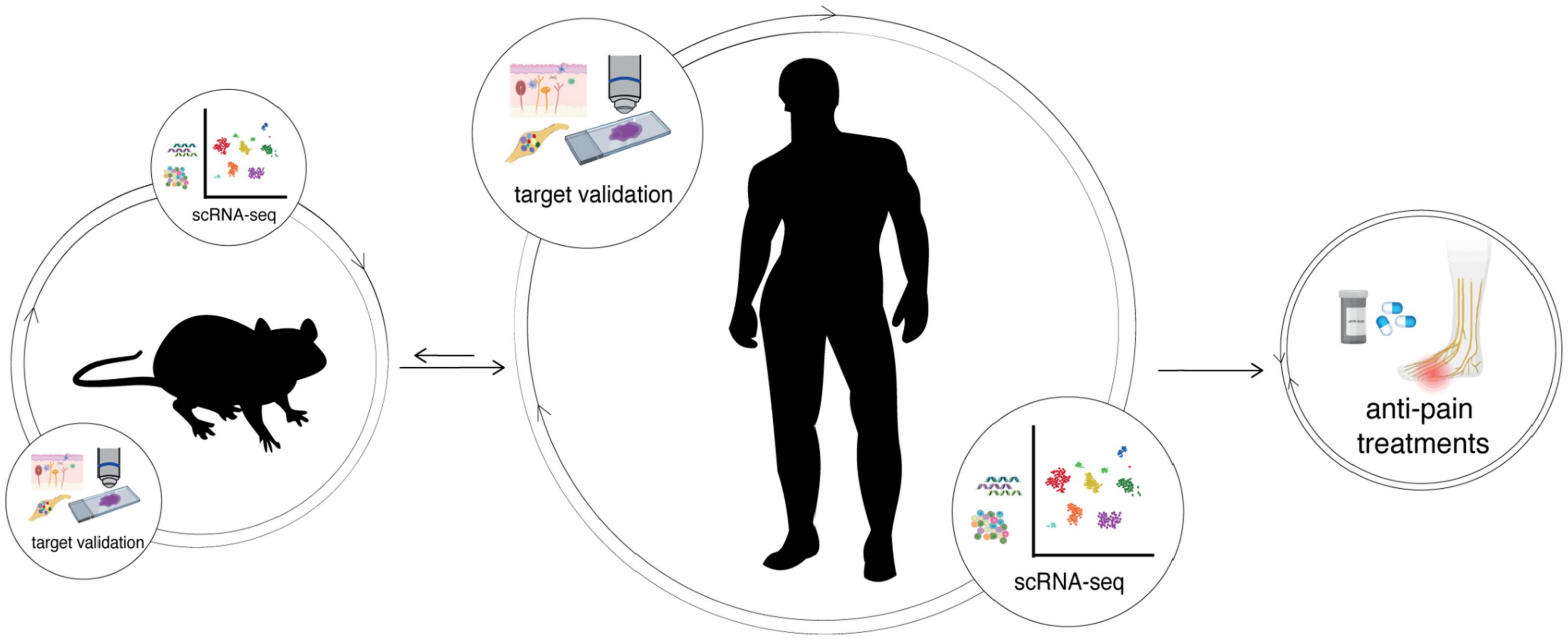
The integration of human and mouse transcriptomes can help bridge translational therapeutic targets between preclinical and clinical studies, characterizing the differences and similarities between both and/or other species. Potential targets identified by an omics-approach always require validation in both rodent and then human tissues, such as in DRGs and the skin. Only after integrating information from different species and performing molecular and functional studies aimed to validate new findings, it is possible to develop effective pain treatments.

An additional critical issue that hinders the translation of our preclinical studies to clinical efficacy is that there exists fundamental molecular and physiological differences in the biology of neuropathic pain between the rodent models compared to humans; in particular, there are clear molecular and electrophysiological differences between rodent and human DRG sensory neuron subtypes (Rostock et al., 2018; Shiers et al., 2020; Jung et al., 2023) (Figure 3). These differences call for the need to use human tissues, such as spinal cord, DRGs, skin biopsies, and iPSCs, in translational pain research, though these tissues have been historically difficult to obtain from patients. However, in recent years the accessibility of functional tissues from organ donors has significantly improved. Sensory neurons derived from organ donors can be isolated and maintained in culture for days to weeks (Valtcheva et al., 2016). These tissues are suitable for genetic, immunohistochemical, RNAscope, imaging, and electrophysiological studies along with multiomic assays and topographic single cell transcriptomics (Ray et al., 2018; North et al., 2019; Shiers et al., 2020; Nguyen et al., 2021; Hall et al., 2022). Indeed, it is now possible to validate candidate mechanisms and novel analgesic targets identified in animal models using human sensory neurons to confirm whether mechanisms of pain sensitization identified in animal models are equivalent in human sensory neurons. Therefore, studies of human tissues are invaluable, and necessary, in prioritizing potential therapeutic targets and refining their targeting strategy (Renthal et al., 2021).

### Single cell transcriptomics

The dawn of transcriptomic analysis has helped to reveal the molecular heterogeneity of DRG neurons and the inconsistency between humans and rodents, providing one insight into the reasons for the failure of much preclinical research.

In addition to functional experiments, next-generation sequencing technology has seen an impressive expansion in recent years, leading to several crucial and valuable discoveries. Currently, there has been an increasing appreciation and focus on the rigorous characterization of individual cell populations within a specific tissue (Tang et al., 2009). Unlike the bulk RNA sequencing approach, single-cell RNA sequencing (scRNA-seq) captures the heterogeneity of cells that comprise tissues, allowing the identification of rare populations that would otherwise not be detected (Tang et al., 2009).

ScRNA-seq has emerged as a valuable tool for investigating gene regulation at high resolution, particularly in the context of detecting functional identity of cell types based on the gene expression profile of single cells within DRGs or other tissues. Multiple groups have conducted scRNA-seq of rodent sensory neurons (Chiu et al., 2014; Sharma et al., 2020), enabling in-depth molecular characterization and facilitating the clustering of DRG neurons and the associated non-neuronal cells into distinct groups to delineate their developmental lineages (Chiu et al., 2014; Sharma et al., 2020). These studies confirmed the anticipated major DRG neuronal types, previously classified based on electrical properties, neuronal size, myelination status, and some established markers (Basbaum et al., 2009). However, single cell transcriptomics allows a comprehensive understanding of the subtypes of DRG sensory neurons (Chiu et al., 2014; Sharma et al., 2020). Unbiased classification of sensory neurons with their extensive molecular profiles enables the understanding of the specific modalities and cellular basis for chronic pain. In this view, we can now understand the changes in gene expression within neuronal subpopulations linked to disease states.

Despite the extensive characterization of the gene expression profiles of injured DRGs using bulk RNA sequencing, there have been gaps in our knowledge of distinguishing which specific cell types is undergoing changes in the transcriptomic profile. Nowadays, scRNA sequencing has delineated the molecular profile of DRG neurons in rodents and primates with chronic pain (Kupari et al., 2021). For instance, Hu et al. performed scRNA-seq on DRG neurons from mice subjected to sciatic nerve transection and their controls (Hu et al., 2016). Notably, an upregulation of genes associated with cell death and changes in pathways linked to neuropathic pain were specifically observed in the non-peptidergic (NP) neuronal subpopulation. This heterogenous transcriptional alteration caused by injury, within distinct neuronal subtypes suggests intrinsic differences in the genetic response to injury between different neuronal subtypes. In addition, the study identified novel targets, like the potassium channels Kcng3 and Kcnn1, in the NP population, which may be important in contributing to pain hypersensitivity through changes in nerve cell excitability (Hu et al., 2016).

scRNA-seq of DRG neurons performed at different time point after spared nerve injury has highlighted the dynamic modifications at single-cell resolution that occur during neuropathic pain (Wang et al., 2021). In addition to the already expected neuronal clusters, other three groups were identified after SNI. These clusters showed higher expression of Atf3, Gal and other nerve-injury regulated genes. Surprisingly, after 24 h, the scRNA-seq analysis showed the presence of a cluster expressing Atf3/Mrgprd (a GPCR), and transcriptomic modification within this cluster led to changes in the phenotype of DRG neurons within 2 days after injury, suggesting that distinct neuron types respond differently to a damage and that, in a injury context, neurons Mrgprd-positive can show high reprogramming capabilities.

Previous studies of scRNA-seq of human DRGs have displayed several significant differences between mouse and human peripheral afferents (Ray et al., 2018; Tavares-Ferreira et al., 2022). An integrative analysis with RNA-seq data of human and mouse DRGs showed large conservation of known DRG and/or nociceptor enriched genes (e.g., *P2XR3* [P2X3 receptor], *SCN10A* [Na_v_1.8], *SCN11A* [Na_v_1.9], *NTRK1* [TrkA], and *MRGPRD* [MRGPRD]) across mouse and human DRGs (Price et al., 2016). However, the relative and co-expression of markers by different subsets was not the same in mice compared to humans (Ray et al., 2018). For instance, CGRP and P2X3R neuronal subpopulation overlap in human lumbar DRGs, but not in mouse DRGs (Shiers et al., 2020). Similarly, several differences were identified in the mRNA expression of transient receptor potential channels, cholinergic receptors, potassium channels, and sodium channels (Shiers et al., 2020). Spatial transcriptomics (10× Genomics Visium) has most recently defined ten neuronal clusters corresponding to low-threshold mechanoreceptors (LTMR) and nociceptors in human DRGs (Tavares-Ferreira et al., 2022). In addition, *TAC1* (encoding SP) and *CALCA* (encoding CGRP) are broadly coexpressed across human nociceptive clusters, in contrast to their separate expression by rodent peptidergic subsets (Tavares-Ferreira et al., 2022). Given the molecular and electrophysiological differences observed in DRG sensory neuron subpopulation across different species, it will be fundamental to validate the mechanisms underlying NeuP discovered to date in mice using human samples (Renthal et al., 2021). By performing single-nuclei RNA sequencing, Jung and colleagues have recently collected and integrated information on the functional profile of sensory neurons from different species (mice, guinea pigs, cynomolgus monkeys and human donors), developing an extensive transcriptome atlas that highlights differences in the expression of molecular modulators involved in sensory function between pre-clinical models and humans, such as *TAFA4* gene (Jung et al., 2023). Moreover, the discrepancy in the abundance of sensory neuron subpopulations across species might also suggest that species-specific biological features need to be taken into constant consideration for developing pain treatment (Jung et al., 2023). Clearly, DRG neurons heterogeneity is crucial for the functional specificity and responses to peripheral stimuli of neuronal subtypes in both mice and humans. We must also acknowledge a few limitations of the scRNA-seq data currently available from donor DRGs including limited clinical informations, small samples size and the lack of longitudinal studies (Bledsoe and Grizzle, 2022). Moving towards the utilization of skin biopsies might overcome some of these limitations.

With decades of essential understanding about nociception and pain from animal models, as well as the availability of cutting-edge tools to investigate genes, cells, and networks at an unprecedented resolution using human samples, we hold the belief that pain research is now in an unique position to change and improve pain treatment.

## Author contributions

PP, JC-D, RM, and DM participated in the writing and editing of this review. All authors contributed to the article and approved the submitted version.

## Conflict of interest

The authors declare that the research was conducted in the absence of any commercial or financial relationships that could be construed as a potential conflict of interest.

## Publisher’s note

All claims expressed in this article are solely those of the authors and do not necessarily represent those of their affiliated organizations, or those of the publisher, the editors and the reviewers. Any product that may be evaluated in this article, or claim that may be made by its manufacturer, is not guaranteed or endorsed by the publisher.
